# Preliminary association of circulating tumor cells (CTCs) pre- and post-AdHER2 dendritic cell vaccination with overall survival in patients with metastatic HER2+ solid tumors

**DOI:** 10.1186/2051-1426-3-S2-P179

**Published:** 2015-11-04

**Authors:** Yusuke Tomita, Jane Trepel, Min-Jung Lee, Sunmin Lee, Jay A Berzofsky, Lauren V Wood

**Affiliations:** 1 NCI/CCR/Developmental Therapeutics Branch, Bethesda, MD, USA; 2 NCI/CCR/Vaccine Branch, Bethesda, MD, USA

## Background

Levels of circulating tumor cells (CTCs) have been associated with overall survival (OS) in metastatic breast [1] and prostate cancer [2]. Here we report the preliminary associations between CTCs and OS in subjects receiving an autologous adenoviral transduced dendritic cell (DC) vaccine expressing human HER2 extracellular (EC) and transmembrane (TM) domains (AdHER2ECTM) in adults with advanced metastatic tumors with 1-3+ HER2 expression. The clinical translation of this vaccine platform was based on animal models that documented regression and cure of large established tumors in syngeneic BALB/c mice using an adenoviral vector vaccine expressing rodent HER2 ECTM mediated by the induction of polyclonal anti-HER2 antibodies [3].

## Methods

In this open label, non-randomized, two part Phase I study (NCT01730118) CTC data are available on 18 subjects (11 F, 7 M, median age 57 years) with HER2+ solid tumors (8 colon cancer, 10 other cancers; IHC 1+ N=3, 2+ N=8, 3+ N=7) that are naïve to HER2-targeted therapies; 16 have received at least 2 doses of an autologous AdHER2 DC vaccine delivered at Weeks 0, 4, 8, 16 and 24. Dose escalation occurred in cohorts of 6 patients utilizing 5x10^6^, 10x10^6^ and 20x 10^6^ viable DCs per vaccine. EpCAM+ CTCs from 10 ml of blood were detected by integrated magnetic pre-enrichment and flow cytometric analysis including assessment of HER2 and CXCR4 expression.

## Results

**Figure 1:**
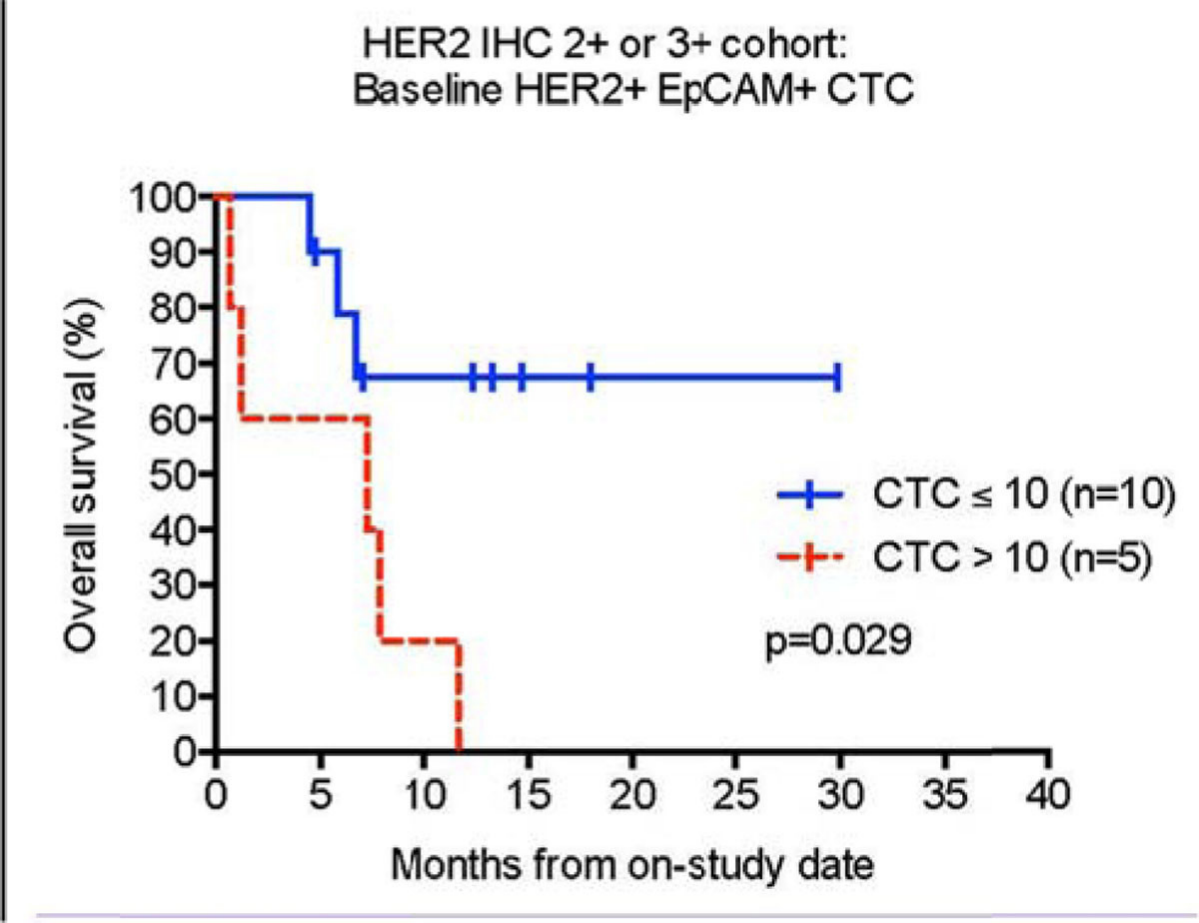


**Figure 2:**
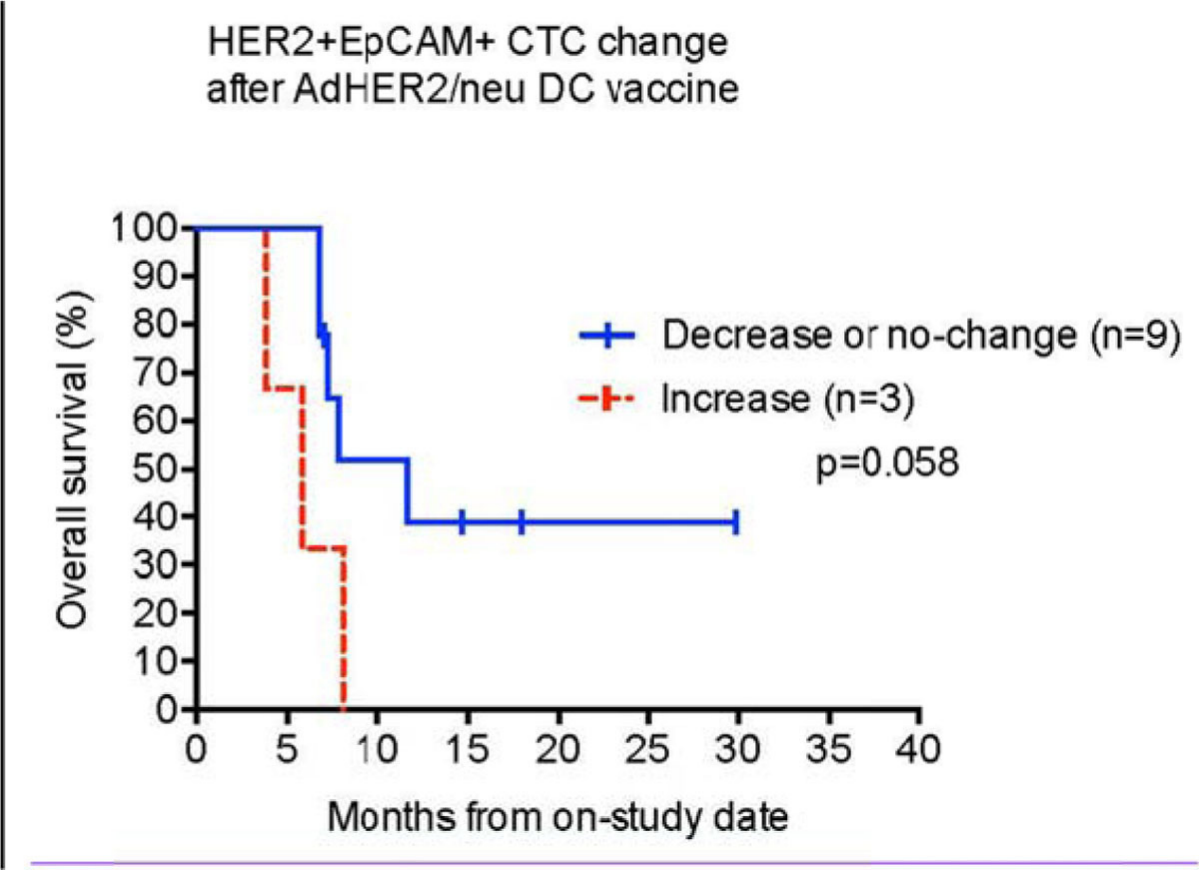


In subjects with HER2 IHC 2+ or 3+ expression (N=15), those with 11 or more HER2+ EpCAM+ CTCs per 10ml of peripheral blood had poorer overall survival (P=0.029) Fig 1. In non-colorectal (CRC) cancer subjects (N=11), patients with 11 or more HER2+ EpCAM+ CTCs or CXCR4+ EpCAM+ CTCs per 10 ml of blood had poorer overall survival (P=0.0046 and P=0.0067, respectively). In this same non-CRC cohort, the HER2+ EpCAM+ CTC count showed a trend toward decrease at study Week 12 in 6 patients status post 3 doses of AdHER2 DC vaccine (P=0.063). Patients with stable or decreasing HER2+ EpCAM+ CTCs following treatment (N=9) exhibited a trend toward better OS (P=0.058) Fig 2.

## Conclusions

In this population of patients with advanced, HER2+ metastatic solid tumors, > 11 HER2+ EpCAM+ CTCs at baseline is associated with poorer OS in patients with HER2 IHC 2+/3+ or non-CRC tumors. Stable or decreasing HER2+ EpCAM+ CTCs following AdHER2 vaccination is associated with a preliminary trend towards prolonged survival. These findings suggest that monitoring HER2+ EpCAM+ CTCs warrants further investigation in patients treated with therapeutic AdHER2 DC vaccination.

## Trial registration

ClinicalTrials.gov identifier NCT01730118.
